# Nutritional and alcoholic contents of cheka: A traditional fermented beverage in Southwestern Ethiopia

**DOI:** 10.1002/fsn3.854

**Published:** 2018-10-25

**Authors:** Belay Binitu Worku, Habtamu Fekadu Gemede, Ashagrie Zewdu Woldegiorgis

**Affiliations:** ^1^ Department of Food Process Engineering and Postharvest Technology Ambo University Ambo Ethiopia; ^2^ Department of Food Technology and Process Engineering Wollega University Addis Ababa Ethiopia; ^3^ Centre for Food Science and Nutrition Addis Ababa University Addis Ababa Ethiopia

**Keywords:** cheka, Dirashe, Konso, traditional beverage

## Abstract

Cheka is a cereal and vegetable‐based beverage which is consumed in Southwestern parts of Ethiopia particularly in Dirashe and Konso. In this study, nine cheka samples were collected from vending houses in Konso and Dirashe districts for the laboratory analysis of the nutritional profile and chemical properties of cheka. The pH and titratable acidity of the samples ranged from 3.53–3.99 and 0.80%–1.11%, respectively. The total solids, crude protein, crude fat, crude fiber, total ash, carbohydrate, and gross energy contents of the samples ranged from 21.05%–26.87%, 3.12–4.44 g/100 g, 1.17–1.81 g/100 g, 0.94–1.27, 0.65–0.93 g/100 g, 14.16–19.03 g/100 g, and 82.04–107.17 Kcal, respectively. The dietary Ca, Fe, and Zn content of the samples were ranged from 8.31–19.60 mg/100 g, 13.94–27.59 mg/100 g and 0.82–1.07 mg/100 g, respectively. The methanol and ethanol contents of the cheka samples ranged from 163.1–2,380 ppm and 3.04%–8.96% v/v, respectively. The findings of this study indicated that cheka has low nutrient content and thus, suggests that people in Konso and Dirashe should not rely on it without eating solid foods as it is almost always diluted with a significant amount of water. In conclusion, the longer fermentation time of cheka resulted in high methanol levels that can present adverse health effects to consumers.

## 1 INTRODUCTION

Fermented beverages have been widely consumed since prehistoric times by people around the world and have formed a traditional part of the human diet (Rose, 1997).In spite of being produced via traditional fermentation, fermented beverages are extremely important in contributing to household nutrition security and in preserving important socio‐cultural practices (FAO, 2012).

Fermented foods and beverages are believed to have enhanced texture, digestibility, and shelf‐life which is attributable to the production of desirable substances such as alcohol (Fellows, 2000; Kohajdova & Karovicova, 2007). However, the presence of toxic substances such as methanol (Fite, Tadesse, Urga, & Seyoum, 1991; Paine & Davan, 2001) in alcoholic beverages puts them in suspicion due to their adverse effects on human health. On the other hand, alcohol (ethanol) in fermented beverages is reported to be the third highest risk for disease and disability, after childhood underweight and unsafe sex (WHO, 2014). Alcohol may have some health benefits if consumed in moderate quantities or otherwise it impairs health and nutrition (Whitney & Rolfes, 2008). Therefore, it is worthwhile to accurately quantify the amount of ethanol and methanol present in unrecorded beverages as they are more likely to be consumed in excess quantities due to their relatively low price compared to factory produced drinks. This will help in reducing health complications and in avoiding other social problems associated with alcohol abuse (Sarah & Mattew, 2012).

Cheka is a cereal and vegetable‐based fermented beverages which is consumed in Southwestern parts of Ethiopia mainly in Dirashe and Konso. People of all ages including infants, pregnant, and lactating women drink cheka. As adults who eat solid foods are considered childish in the communities, it is cheka that is being consumed all day long and from observation an adult man on average drinks up to 8 L of cheka per day. Several works have been done on Ethiopian fermented beverages such as *tella* (Desta, 1977; Yohannes, Fekadu, & Khalid, 2013), *tej* (Yohannes et al., 2013), *arake* (Yohannes et al., 2013), *shamita* (Ashenafi & Mehari, 1995), *borde* (Abegaz, Beyene, Langsrud, & Judith, 2002a), and *kerebo* (Rashid, 2013a). However, no work has been done on any aspect of cheka until present. Therefore, this study was intended to (a) determine the nutritional content of cheka and (b) detect and quantify the alcoholic contents of cheka using a gas chromatographic technique.

## 2 MATERIALS AND METHODS

### 2.1 Sample collection

Nine cheka samples were collected using screw‐cap plastic containers (1 L each) from vending houses at three localities for analysis of their chemical properties, nutritional, and alcohol contents. All the samples were collected purposively while considering the processing techniques and duration of fermentation. The samples were coded with the first letter of the site and numbers in the order they were collected and were transported to the laboratory of the Centre for Food Science and Nutrition at Addis Ababa University using an icebox. In addition, one sample of cheka was prepared by the investigator in the laboratory following the Konso preparation method using yellow maize, sorghum grains, and barley obtained from local markets of Addis Ababa.

### 2.2 Sample preparation

After transportation of the samples, moisture analysis and determinations of pH and titratable acidity were immediately carried out in the laboratory. For convenience of proximate analysis, the samples were lyophilized and were packed in polyethylene plastic bags to be stored in dry place. But the methanol and ethanol contents of the cheka samples were determined in liquid form.

### 2.3 pH and titratable acidity

The pH of the samples was measured by dipping the glass electrode of a digital pH meter into 10 ml of the sample after blending with distilled water at a 1:1 ratio into thick slurry as described in Abegaz, Beyene, Langsrud, and Judit (2002b). For the determination of total titratable acidity, about 10 ml of cheka samples were added into beakers (50 ml) and titrated against 0.1 N standard solution of NaOH after adding 3 drops of 1% phenolphthalein indicator (Byaruhanga, 1998; cited in Rashid, 2013b). The percent of lactic acid present in the sample was calculated using the following formula.

$$\%\;\text{Lactic acid}\;\left(\text{wt}/v\right)=\frac{N\;\times \;V_{\mathrm{NaOH}}\;\times \;\text{Eq}.\;\text{wt}\;\times \;100}{V_{s}\;\left(\text{ml}\right)\;\times \;1,000}$$

where; N = normality of titrant (mEq/ml), *V*_NaOH_ = Volume of titrant (ml), Eq. wt = Equivalent weight of predominant acid (mg/mEq which is 90.08 for lactic acid), *V*_s_ = Volume of sample (ml) and 1,000 = factor relating mg to grams.

### 2.4 Proximate composition analysis

The total solids, crude protein, crude fat, crude fiber, and total ash contents of the samples were analyzed according to the AOAC (2000) methods and ASEAN manual of food analysis (2011). The utilizable carbohydrate content was determined by subtracting the sum of the percentages moisture, crude protein, crude fat, crude fiber, and total ash from 100%. The calorific value of the cheka samples were determined by calculation from protein, fat, and carbohydrate contents using Atwater's conversion factors (Guyot, Rochette, & Treche, 2007).

### 2.5 Mineral analysis

Mineral composition of the samples was determined according to methods recommended by Association of Official Analytical Chemists (AOAC, 2000) and ASEAN manual of food Analysis (ASEAN FOODS, 2011). Ca, Fe, and Zn contents of the cheka samples were determined by atomic absorption spectrophotometer (AA‐7000 Series, Shimadzu Scientific Instruments, Kyoto, Japan) following standard procedures.

### 2.6 Alcohol analysis

The ethanol and methanol contents of the cheka samples were determined by a gas chromatography method (GC 2010 Plus, Shimadzu Scientific Instruments, Kyoto, Japan). The samples were prepared according to the method developed by Tangerman (1997). The gas chromatographic conditions were set following the method developed by Wang, Choong, Su, and Lee (2003).

#### 2.6.1 Sample preparation

About 10 ml of cheka was measured in a plastic test tube and ultracentrifuged for 2 hr at 4°C and 30,000 *g*. About 5 ml of the cheka supernatant was carefully removed and transferred into a conical polypropylene tube and centrifuged for 1 min at 10,000 *g*. The clear supernatants were stored in a refrigerator and later used for the chromatographic analysis. For analysis, the supernatant was filtered through micro filter (0.45 m PTFE membrane) into vial (1.8 ml).

#### 2.6.2 Preparation of standard solution

Series of solutions ranging from 0.1%–15% and 0.005%–2% were prepared to make a standard calibration curves for ethanol and methanol, respectively. The limit of detection (LOD) and limit of quantitation (LOQ) were determined by multiplying the standard deviation obtained from 10 repeated injections of solutions with low concentrations of 0.005% (methanol) and 0.1% (ethanol) by 3 and 10, respectively. The recovery test was done by spiking a known amount of methanol (0.5 and 5 ml of 1% solution which were equivalent to 5 and 50 μl, respectively) and ethanol (0.5 and 5 ml of 5% solutions which were equivalent to 25 and 250 μl) into 10 ml of cheka.

#### 2.6.3 Gas chromatography conditions

Ethanol and methanol analysis was performed using Shimadzu GC‐2010 Plus gas chromatograph which was equipped with an FID detector, AOC‐20i+S autosampler and with a GC solution software for data handling system. The length, inner diameter, and film thickness of the column were 30 m, 0.25 mm and 0.25 μm, respectively. The flow rates of H_2_ and N_2_ gas were set at 30 and 300 ml/min, respectively. The temperatures of the FID detector and the injection port were set at 300 and 225°C, respectively. The column temperature was set initially at 45°C for 2 min and then ramped at a rate of 45°C/min to the final 245°C. The injection volume was limited to 1.0 μl using split injection mode.

### 2.7 Data analysis

Determinations were done in duplicates for the need of statistical analysis. Data were computed using SPSS (version 20) statistical software packages. Data were expressed as mean ± standard deviations of the replicate determinations. One‐way analysis of variance (ANOVA) was used to study the significant difference between the samples with respect to the studied parameters. Least significant difference (LSD) at *p* < 0.05 was used to determine which means were significantly different.

## 3 RESULTS AND DISCUSSION

### 3.1 pH and titratable acidity

pH and titratable acidity are one of the most important parameters that determine the flavor and shelf‐life of a food product. In this study, the pH and titratable acidity of the cheka samples ranged from 3.53–3.99 and 0.77%–1.11%, respectively (Table 1). Although the samples coded as, K2 (3.53) and K3 (3.55) had the lowest pH value, they did not significantly differ from samples coded as K1 (3.58) and G3 (3.68). The samples S2 (3.99), S3 (3.89), and G1 (3.95) had the highest pH value and no significant difference (*p* > 0.05) was observed among them. K1 (1.11%) had the highest percent titratable acidity followed by K2 (1.07%) and K3 (1.01%). No significant difference was found between S1 (0.91%), S2 (0.91%), S3 (0.89%), G2 (0.90%), and G3 (0.86%). The cheka produced in the laboratory had pH of 3.91 which was comparable with the pH of the samples S2, G1, and S3, but it had significantly (*p* < 0.05) lower percent titratable acidity (0.77%) than most of the collected samples.

**Table 1:** The pH and titratable acidity of the cheka samples

Sample code	pH value	% Titratable acidity
K1	3.58 ± 0.08^cd^	1.11 ± 0.03^a^
K2	3.53 ± 0.05^d^	1.07 ± 0.01^a^
K3	3.55 ± 002^d^	1.01 ± 0.01^b^
S1	3.76 ± 0.08^b^	0.91 ± 0.03^c^
S2	3.99 ± 0.04^a^	0.91 ± 0.01^c^
S3	3.89 ± 0.03^a^	0.89 ± 0.02^c^
G1	3.95 ± 0.06^a^	0.80 ± 0.03^d^
G2	3.72 ± 0.05^b^	0.90 ± 0.03^c^
G3	3.68 ± 0.02^bc^	0.86 ± 0.01^c^
CL	3.91 ± 0.17^a^	0.77 ± 011^d^

*Notes*

All the values are mean ± standard deviation of duplicate determinations. Means within the same column with different letter superscripts indicate significant differences (*p* < 0.05).

CL: cheka prepared in the laboratory; K: Konso; S: Shelele.

Generally, samples from Konso had low pH values and samples from Shelele had the highest pH values. The variation observed between the samples in their pH value could be due to the differences in fermentation time and type of cereals utilized. Based on the finding of this study, cheka had low mean pH value of 3.75, which is lower than that of other Ethiopian traditional beverages such as *tella* (Yohannes et al., 2013), *tej* (Gizaw, 2006; Yohannes et al., 2013), *borde* (Abegaz et al., 2002b), *shamita* (Ashenafi & Mehari, 1995), *kerebo* (Rashid, 2013a), and arake (Yohannes et al., 2013). On the other hand, ready to consume cheka had titratable acidity comparable to *borde* (Abegaz et al., 2002b) but lower titratable acidity than *kerebo* (Rashid, 2013a). Since the pH of the samples were measured once immediately after they had been brought to the laboratory, the investigators believe that the pH of cheka can even be lower than the reported values in this study if determined over a period of time until it turns not safe to consume. Evidences point out that the low pH of beverages can rob calcium in skeletal systems and lead to dental caries and osteoporosis (Cairns, Watson, Creanor, & Foye, 2002; Mettler, Carmen, & Paolo, 2006). Therefore, if the pH of cheka drops below the reported values (especially after 2 days of consumption), it can cause harms to the skeletal system of the consumers.

### 3.2 Proximate composition

In this study, the total solids content of cheka varied between 21.05% (G2) and 26.87% (S3). There were no significant differences (*p* > 0.05) in the total solids contents of K1 (25.47%), K2 (22.44%), K3 (22.67%), S1 (23.83%), S2 (22.81), G2 (23.48%), and G3 (23.11%). It was found that the crude protein and fat contents of the cheka samples ranged from 3.02 to 4.44 g/100 g and 1.17 to 1.81 g/100 g, respectively (Table 2). Two of the samples collected from Gidole town (G1 and G3) had significantly (*p* < 0.05) high protein content of 4.44 g/100 g and 4.40 g/100 g, in that order and were followed by the sample coded as S3 (4.19 g/100 g) which was collected from Shelele kebele. On the other hand, the cheka sample prepared in the laboratory had a significantly low crude protein content (3.02 g/100 g) when compared to the samples S3 (4.19 g/100 g), G1 (4.44 g/100 g), and G3 (4.40 g/100 g).

**Table 2:** Proximate composition of the cheka samples

Sample code	% Total solids	Crude protein	Crude fat	Crude fiber	Total ash	Carbohydrate	Gross energy (Kcal)
K1	25.47 ± 0.35^ab^	3.53 ± 0.02^c^	1.81 ± 0.08^a^	1.07 ± 0.02^a^	0.82 ± 0.07^ab^	18.24 ± 0.33^ab^	95.59 ± 2.17^abc^
K2	22.44 ± 1.68^bc^	3.38 ± 0.19^c^	1.44 ± 0.12^bc^	0.94 ± 0.09^a^	0.65 ± 0.03^b^	16.02 ± 1.24^bcd^	90.60 ± 5.23^bc^
K3	22.67 ± 1.28^abc^	3.12 ± 0.14^c^	1.42 ± 0.05^bc^	1.00 ± 0.04^a^	0.76 ± 0.03^ab^	17.57 ± 1.08^abc^	107.17 ± 1.03^a^
S1	23.83 ± 0.55^abc^	3.51 ± 0.14^c^	1.42 ± 0.01^bc^	1.00 ± 0.02^a^	0.71 ± 0.01^b^	17.19 ± 0.36^abc^	103.17 ± 2.00^ab^
S2	22.81 ± 1.33^bc^	3.53 ± 0.15^c^	1.36 ± 0.11^cd^	1.19 ± 0.05^a^	0.68 ± 0.04^b^	16.05 ± 0.98^bcd^	90.60 ± 6.85^bc^
S3	26.87 ± 0.31^a^	4.19 ± 0.09^ab^	1.59 ± 0.01^b^	1.14 ± 0.01^a^	0.93 ± 0.04^a^	19.03 ± 0.37^a^	95.60 ± 5.34^abc^
G1	23.48 ± 2.37^abc^	4.44 ± 0.51^a^	1.35 ± 0.09^cd^	1.24 ± 0.13^a^	0.76 ± 0.15^ab^	15.70 ± 1.48^cd^	92.69 ± 8.81^bc^
G2	21.05 ± 2.40^bc^	3.71 ± 0.34^bc^	1.17 ± 0.16^d^	1.27 ± 0.35^a^	0.73 ± 0.06^b^	14.16 ± 1.50^d^	82.04 ± 8.75^c^
G3	23.11 ± 1.26^abc^	4.40 ± 0.18^a^	1.37 ± 0.04^cd^	1.24 ± 0.07^a^	0.74 ± 0.11^b^	15.36 ± 0.86^cd^	91.38 ± 4.50^bc^
CL	22.57 ± 2.78^bc^	3.02 ± 0.43^c^	1.49 ± 0.10^bc^	1.19 ± 0.16^a^	0.67 ± 0.08^b^	16.21 ± 2.02^bcd^	90.28 ± 10.68^bc^

*Notes*

All the values are mean ± standard deviation of duplicate determinations. Means within the same column with different letter superscripts indicate significant differences (*p* < 0.05).

CL: cheka prepared in the laboratory; K: Konso; S: Shelele.

The cheka samples had high mean protein content (15.68 g/100 g on dry matter basis) when compared to *borde* and *shamita* which have about 9.55 g/100 g and 10.37 g/100 g protein on dry basis, respectively (Ashenafi & Mehari, 1995). Sample K1 (1.81 g/100 g) had the highest fat content than all other samples whereas samples G1 (1.35 g/100 g), G2 (1.17 g/100 g), and G3 (1.37 g/100 g) had the least fat contents. But statistical analysis of the result showed no significant (*p* > 0.05) variation between the samples coded as K2 (1.44 g/100 g), K3 (1.42 g/100 g), and S1 (1.42 g/100 g). The crude fat content of CL (1.49 g/100 g) was significantly lower than that of K1 (1.81 g/100 g) but higher than the rest of the sample. Cheka samples had a mean fat content of 6.11 g/100 g on dry matter basis which is comparable with the fat content of *borde* (6.88 g/100 g), but more fat content than *shamita* (3.46 g/100 g) (Ashenafi & Mehari, 1995).

The total carbohydrate and fiber content of the cheka samples ranged from 14.16 to 19.03 g/100 g and 0.94 to 1.27 g/100 g. The results of this study showed that sample K3 (19.03 g/100 g) had high amount of carbohydrate followed by K1 (18.24 g/100 g), K (17.57 g/100 g), and S1 (17.19 g/100 g) in that order and no significant difference (*p* > 0.05) existed between these four samples. Even though there were no significant variations between the samples in their fiber content, samples from Gidole and Shelele kebele had relatively higher fiber contents than samples collected from Konso. The crude fiber content of cheka prepared in the laboratory was comparable with that of the samples collected from the study areas. On the other hand, the gross energy content of cheka samples varied from 82.04 Kcal/100 g for G2 to 107.17 Kcal/100 g for K3. Significant variations were observed among the samples and generally samples from Gidole (G1, G2, and G3) had low gross energy. The ash content of cheka samples ranged from 0.65–0.93 g/100 g. The sample coded as S3 had the higher ash content (0.93 g/100 g) than samples K2 (0.65 g/100 g), S1 (0.71 g/100 g), S2 (0.68 g/100 g), G2 (0.73 g/100 g), and G3 (0.74 g/100 g), but it did not significantly (*p* > 0.05) differ from K1 (0.82), K3 (0.76 g/100 g), and G1 (0.76 g/100 g). No significant variation was observed between CL (0.67 g/100 g) and most of the samples collected from the study areas in their ash contents except S3 (0.93 g/100 g). The sample represented by K2 had the least ash content though it did not significantly differ from most of the samples except S3. The cheka samples analyzed in this research had lower average ash content on dry matter basis (3.15 g/100 g dry basis) than *borde* and *shamita* which have mean ash contents of 6.85 g/100 g and 3.66 g/100 g, respectively (Ashenafi & Mehari, 1995). The variations observed in the proximate composition of the samples greatly reflects the differences in some of the raw materials utilized for cheka preparation and the fermentation time as well.

### 3.3 Mineral composition

**Figure 1:**
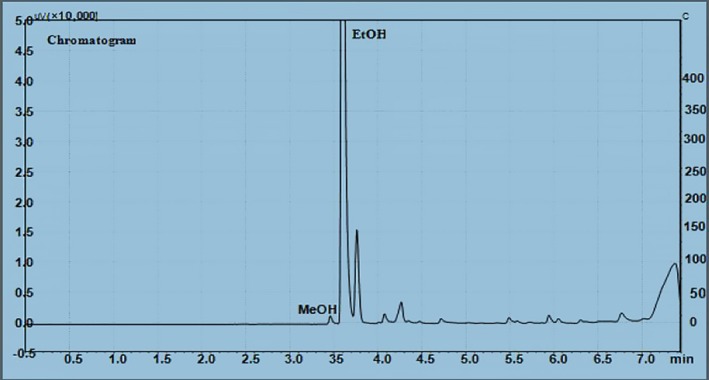
Chromatogram of methanol and ethanol detected from cheka sample

In the present study, one of the most important mineral elements such as calcium, iron, and zinc were analyzed. As presented in Table 3, the cheka samples contained Ca, Fe, and Zn levels ranged from 8.31–19.60 mg/100 g, 13.94–27.59 mg/100 g, and 0.82–1.07 mg/100 g, respectively (Table 3). Statistical analysis of the result showed significant (*p* < 0.05) variations in the contents of all the three minerals. Samples collected from Gidole (G1, G2, and G3) and Shelele (S1, S2, and S3) had significantly higher calcium than those collected from Karat (Figure 1). One of the samples from Karat, K1 (1.07 mg/100 g), had the highest zinc content followed by samples K3 and S1, and S3 all having about 0.95 mg/100 g. The cheka sample produced in the laboratory had comparatively low mineral content than most of the collected samples. The relatively low mineral contents of the cheka samples collected from Karat could be due to the fact that brewers in Konso are highly market oriented and utilize only grains because it helps them produce cheka frequently.

**Table 3:** Mineral composition of the cheka samples (mg/100 g)

Sample code	Ca (mg/100 g)	Fe	Zn
K1	10.90 ± 1.93^e^	18.96 ± 0.73^cd^	1.07 ± 0.09^a^
K2	8.31 ± 1.45^e^	13.94 ± 0.87^e^	0.86 ± 0.09^c^
K3	14.54 ± 0.25^cd^	15.29 ± 1.43^e^	0.95 ± 0.04^abc^
S1	18.26 ± 0.70^ab^	24.14 ± 0.93^b^	0.95 ± 0.01^abc^
S2	15.33 ± 0.04^bc^	16.32 ± 2.57^de^	0.86 ± 0.03^c^
S3	18.31 ± 0.19^ab^	27.59 ± 1.36^a^	0.95 ± 0.04^abc^
G1	16.52 ± 1.11^abc^	16.49 ± 1.73^de^	0.82 ± 0.08^c^
G2	19.60 ± 3.33^a^	21.22 ± 2.09^bc^	0.90 ± 0.05^bc^
G3	11.14 ± 0.44^de^	14.41 ± 0.81^e^	1.02 ± 0.04^ab^
CL	14.38 ± 1.03^e^	14.41 ± 0.81^e^	0.79 ± 011^c^

*Notes*

All the values are mean ± standard deviation of duplicate determinations. Means within the same column with different letter superscripts indicate significant differences (*p* < 0.05).

CL: cheka prepared in the laboratory; K: Konso; S: Shelele.

### 3.4 Alcoholic contents

#### 3.4.1 Methanol content

The methanol content of the cheka samples ranged from 0.0163% to 0.2385% (v/v) (Table 4). The sample S3 (2,384.4 ppm) had significantly (*p* < 0.05) high methanol content than the remaining samples and was followed by K1 (0.1630% v/v) and S2 (0.1361% v/v). The lowest methanol content was recorded for sample S1 (0.0163% v/v), but it did not significantly differ from other samples except K1, S2, and S3. Generally, samples from Shelele contained significantly high amount of methanol. The reason for high methanol content in Dirashe could be due to the longer fermentation time(more than a month) that allows more pectins in the product to be degraded by pectinase enzymes into methanol (Singkong, Rattanapun, & Kaweewong, 2012). As presented in Table 4, the high methanol content of some of the samples correspond to the high ethanol content.

**Table 4:** Methanol and ethanol content of the cheka samples

Sample code	Methanol content (% v/v)	Ethanol content (% v/v)
K1	0.1630 ± 0.042^b^	8.96 ± 0.35^a^
K2	0.0178 ± 0.006^c^	5.65 ± 3.2^ab^
K3	0.0328 ± 0.020^c^	5.84 ± 1.14^ab^
S1	0.0163 ± 0.005^c^	3.05 ± 0.29^b^
S2	0.1361 ± 0.022^b^	7.38 ± 1.39^ab^
S3	0.2385 ± 0.024^a^	8.02 ± 1.70^a^
G1	0.0369 ± 0.048^c^	3.12 ± 0.29^b^
G2	0.0595 ± 0.004^c^	5.00 ± 3.52^ab^
G3	0.0288 ± 0.001^c^	5.63 ± 1.47^ab^
CL	0.0249 ± 0.004^c^	7.06 ± 1.86^ab^

*Notes*

All the values are mean ± standard deviation of duplicate determinations. Means within the same column with different letter superscripts indicate significant differences (*p* < 0.05).

CL: cheka prepared in the laboratory: K: Konso; S: Shelele.

The amount of methanol reported in this study for most samples is much higher than the methanol contents reported for *tella* (32.37 ppm) and *tej* (45.67 ppm), and *arake* (320.87 ppm) (Fite et al., 1991). The samples coded as K1, S2 and S3 had much higher methanol content than the specifications for maximum methanol and wine (ESA, 2013). All samples had methanol content higher than the maximum limit specified by East African Standards for gin (EAS, 2013) and also half of the samples had more methanol content than the limit set by EU regulation (cited in Paine & Davan, 2001). This shows that there might be the possibility of methanol toxicity in the study localities where the fermentation time is longer.

#### 3.4.2 Ethanol content

The alcohol content of the cheka samples analyzed varied between 3.05% v/v and 8.96% v/v (Table 4). One of the samples from Konso (K1) had the highest ethanol content (8.96% v/v) followed by the sample S3 (8.02% v/v), which was collected from Shelele. Significant variation was observed among some of the samples in their ethanol contents. Sample S1 (3.02% v/v) had the least ethanol content, but it did not significantly differ from most samples except samples K1 (8.96% v/v) and S3 (8.02% v/v). This variation could be due to the differences in the duration of fermentation time and also the introduction of unequal amount of fresh flour during cheka production. Since cheka fermentation is mediated by natural microbes from raw materials and equipment, the yeast strains and their load may vary which contributes to the observed variation in ethanol content. In the case of Konso cheka preparation, there is almost no introduction of fresh flour once the fermentation started, but fresh flour must be added into Dirashe cheka 1 day in advance of cooking the dough balls. Based on this finding, the ethanol content of the cheka samples was comparable with that of *tella* (2.5%–14.52%) and *tej* (6.2%–14%) (Desta, 1977; Gizaw, 2006; Sahle & Gashe, 1991; Yohannes et al., 2013). However, the alcohol content of the cheka samples was much lower than that of *arake* which has alcohol content as high as 48% v/v (Gizaw, 2006).

## 4 CONCLUSION

As people in the study areas use cheka after diluting with sufficient water due to its thick consistency, high alcohol content and low acidity, the crude protein, fat, fiber, carbohydrate, ash, and energy content of the cheka can be lower than the values reported in this study. This necessarily indicates that adults who rely on cheka are still needy for the consumption of other solid foods in order to meet their daily nutrient and energy requirements. The cheka samples with longer fermentation time had higher methanol contents to levels that can pose adverse effects on the health of consumers. In addition, cheka had high ethanol levels that might be not safe for some individuals including pregnant and lactating women, children and adolescents. From food safety point of view, investigations on the mechanism of cheka production and means to avoid unpleasant contents are necessary.

## CONFLICT OF INTEREST

The authors declare that there is no conflict of interest.

## ETHICAL STATEMENT

This study did not involve any human or animal testing.
